# Hospital Meetings, &c.

**Published:** 1898-02-19

**Authors:** 


### HOSPITAL MEETINGS, &C.

### THE ROYAL NATIONAL HOSPITAL FOR CONSUMPTION.

At the annual meeting of this institution, held on Wednesday, 9th inst., the Attorney-General (Sir Richard Webster) presided, and there was an influential attendance, including Mr. Neale F. Horne (deputy chairman), the Master of St. Katharine's, Mr. H. C. Richards, M.P., Dr. Sinclair Coghill, Mr. P. B. Burgoyne (treasurer), and Mr. Ernest Morgan (secretary).

The report presented showed that the year 1897 had been one of progress, for while the liberal support given to the hospital enabled the board to keep every bed occupied, a new block of three houses has been commenced, which will afford accommodation for twenty-one additional patients, and bring the total number of beds available up to 155. Considering that there are often upwards of 150 accepted candidates awaiting admission, the increase is urgently needed, and Sir Richard Webster, in dwelling upon the subject, observed that eminent medical men attach great importance to the early treatment of consumption. "If we can shorten the time of waiting for admission," added Sir Richard, "I am sure we shall be doing good work." The new block is to be completed early next year, and the plans include every modern scientific and sanitary appliance. Verandahs will be provided on each floor, facing south, and will be sufficiently wide to admit of beds being wheeled through the French casements when required, so that as far as possible the open air and sunshine treatment may, in suitable cases, be carried out.

The financial statement indicated that for the year the ordinary receipts amounted to £10,980 18s., and the expenditure to £11,346 1s. 10d. The receipts, which include £262 10s. from the Prince of Wales's Fund, show a decrease of £851 as compared with 1896—the dinner year—when the exceptional sum of £3,733 was received in donations. The extraordinary receipts consisted of the treasurer's gift of 2,000 guineas for a house. No legacies were paid over during the year, but notices of some have been received, and the amounts will be paid during 1898.

So far as patients are concerned, 1897 was a record year. The number treated during the twelve months was 848, an increase of 38 over 1896, which up to that time was the largest in the hospital's history. This increase is accounted for by the expedition with which the annual cleaning was carried out, and the prompt manner in which vacancies were filled. The duration of residence averaged 62·5 days, practically the same as in the previous year. The patients who completed their term of treatment numbered 717, and the results are tabulated as follows: Very much improved, 107; much improved, 198; improved, 253; in *statu quo*, 81; worse, 43; died, 32. The death-rate, calculated from the 848 patients, was 3·7 per cent.

The treasurer and retiring members of the committee having been re-elected, and Sir E. Montague Nelson added to the committee, votes of thanks were accorded to the officials and chairman, and the annual meeting then terminated.

### LONDON FEVER HOSPITAL.

There was a small attendance at the annual meeting of the London Fever Hospital, held at the Hotel Victoria on Friday, the 11th inst. Lord Balfour of Burleigh, who presided, described the past year as an uneventful one in the hospital's history. The new ward block which was opened early in the year has been fully occupied, but in consequence of the financial position of the institution the process of rebuilding the hospital has been suspended. The old wing is not in a fit state of repair to receive patients, and as a consequence the number of cases the hospital can accommodate is temporarily reduced. When the rebuilding is complete the hospital will, according to Mr. John Hamer, who presented the Finance Committee's report, be the finest fever hospital in the world. "But," said Mr. Hamer, "we must not trench on our annual subscriptions; we must build only as far as our legacy funds will permit us." For 1897 the ordinary receipts amounted to £11,342 3s. 10d., and the ordinary expenditure to £10,523 19s. 3d. The extraordinary receipts aggregated £4,039 4s. 7d., and the extraordinary expenditure to £4,194 14s. 10d. A comparison with the previous year shows that for 1897 the legacies amounted to £3,752, against £1,572; the annual subscriptions to £4,401, against £4,319, and the donations to £1,631, against £2,384. The falling off of £753 in the donations was more than compensated for by the grant of £875 from the Prince of Wales's Fund. But Lord Balfour assured the meeting that a hint had been received to the effect that this must not be counted upon for the future, and he therefore cordially supported the appeal made by Mr. Hamer that the friends of the hospital should come forward to its support. Lord Balfour also commented on the fact that the grant from the Hospital Saturday Fund amounted only to £25. This he declared to be a small sum considering that 360 of their patients were the very people who contributed or ought to be contributing to the Fund. During 1897 579 patients were admitted, and in addition there were 128 under treatment at the beginning of the year. The total number coming under treatment was, therefore, 707, compared with 874 during 1896. Of the patients admitted there were 431 with scarlet fever, 58 with German measles, 45 with diphtheria, and 31 sent in under medical certificates of infectious fever, but who were suffering from various non-infectious complaints. The total number of scarlet fever cases under treatment was 530, against 720 in 1896. It was for the most part of a mild type, the mortality being only 1·7 per cent. The cases of measles numbered 13, all of which were discharged cured except two, who were convalescing in the hospital at the end of the year. Fifty-eight cases of German measles were treated; 56 were discharged cured, and two remained in the hospital. As regards diphtheria, only such cases as presented the bacillus of Loeffler were included under this head. There were 62 in all, 17 of which were in the hospital at the beginning of the year. Six died, 50 were discharged cured, and 6 re-remained on December 31st. Of the officials two nurses contracted diphtheria, one nurse and one wardmaid scarlet fever, and one nurse measles, but all recovered. The decrease in the number of scarlet fever cases alluded to above is due first to the reduction in the accommodation resulting from the disuse of the old ward, and in the second place to the fact of measles having been introduced into one of the main scarlet fever wards by a patient incubating the disease when suffering from scarlet fever. This necessitated the non-admission of any more cases of any kind into the ward until it could be gradually cleared of the patients it contained and thoroughly disinfected. The committee have entered upon a new departure in appending to the report a statement of the social position of the patients. Of the 579, 95 were admitted free by governor's order; 7 free, or by payment of reduced fee; 436 by payment of fee of three guineas; and 41 to private wards. In the classification, 54 are described as belonging to professions. There were 37 nurses, 47 domestic servants, 186 employés, clerks, and families, 176 tradesmen and families, 6 from schools, 5 from other hospitals, 13 from charitable institutions, 7 policemen and families, 6 soldiers, sailors, and families; while 42 others were accommodated in private rooms. The meeting having adopted the report, re-elected the President and also the committee, with the addition of Brigade-Surgeon Chappell, M.D., Mr. G. Stapylton Barnes, and Mr. J. Bentinck. Votes of thanks terminated the proceedings.

### ST. MARK'S HOSPITAL.

The annual meeting of the St. Mark's Hospital for Fistula, City Road, E.C., was held on Thursday, 10th inst., at the Mansion House, under the presidency of the Lord Mayor. It was stated in the report that nearly half the beds were closed for want of funds, and the committee hoped that renewed efforts would be made to raise sufficient funds to justify them in opening the whole of the wards this year. Three hundred and nine in-patients and 886 out-patients were treated during the year. The report was adopted on the motion of the Lord Mayor, seconded by Mr. Alfred Cooper. Three members of the Committee of Management were elected. Votes of thanks were given to the various officers, and the meeting terminated.

### HOSTEL OF ST. LUKE.

The Archbishop of Canterbury presided over the annual meeting of the Hostel of St. Luke, held at the Church House on Thursday, February 10th. There was a very large attendance, including the Bishop of Bristol, the Bishop of St. Albans, Canon Barker, Chancellor P. V. Smith, and Canon Utterton. The report submitted shows that the income for the year, inclusive of £97 13s. 5d. brought forward from 1896, amounted to £1,762 13s. 5d., and the expenditure to £1,588 19s. 6d. The principal sources of income were: Subscriptions and donations, £723; offertories, £566; and paying patients, £317. The Council attribute the falling off in subscriptions to the Jubilee, and state that but for the result of a special appeal by Canon Utterton it might have been necessary to have recourse to the reserve fund. As it is they have been unable to make any investment of surplus income. His Grace, in the course of a brief address, remarked that the report did not give the number of patients treated, and the pressure had been at times so great that sleeping accommodation had to be found outside the hostel for some of the nurses. They hoped, however, in a short time to be able to remove into a larger and better building. His Grace added that he had made many inquiries about the institution, and found unanimous expressions of praise for the work it accomplished. Another speaker, the Bishop of Bristol, suggested that it would be well if the number of patients was included in the report in future, and Canon Barker remarked that the hostel was of such value to the clergy and their families that it ought to be more widely known. Medical and surgical treatment, nursing, board, and attendance were provided free in necessitous cases, and no distinction was made between free and paying patients. Then again, they found at the hostel the comfort and privacy of a home, and friends of the patients were admitted daily, a most valuable boon. The council were re-elected with the addition of the Rev. R. H. Sinclair, St. Anselm's, Davies Street; Rev. J. Miles, St. Catharine Cree; Rev. W. T. Houldsworth, St. Andrew's, Well Street; and the Rev. Gerald Harcourt, All Saints, Battersea. A resolution having been adopted declaring that the hostel deserves the cordial support of the clergy and laity, the meeting closed with the usual votes of thanks.

### THE CLAPHAM MATERNITY HOSPITAL.

The annual meeting of the Clapham Maternity Hospital took place on February 11th, at half-past three, at the hospital, 41 and 43, Jeffrey's Road. The Hon. Mrs. Talbot took the chair. The meeting was well attended. Mrs. Talbot recommended the hospital to the support of those present on five pleas: That it was a woman's work for women; that it was of special use to poor married women, who regarded the short time spent there as one of physical and spiritual rest and refreshment, and who strongly appreciated their removal from the vulgar environment amidst which the important events of life take place amongst the poor; that it was an effectual agent in rescue work; that it was a power on behalf of temperance; and, lastly, that it was a valuable training school, not only for nurses, but also for medical women, who were thus enabled to study midwifery apart from male students. The hospital is solvent; it closes the year with a balance of £20 to the good. In spite of a long and severe septic case, which has taxed the resources of the hospital severely (and which was successfully isolated and recovered), only two deaths occurred, one caused by pneumatic embolism, and one by rupture of the womb. The hospital now issues an appeal for £12,000. The work is increasing in extent, in usefulness, and in the regard in which it is held by the women who benefit. The difficulties of isolating a septic case, and the impossibility of admitting a septic out-patient who requires the care afforded by constant watchfulness and skill, are greatly felt and deprecated. It is also desirable that conveniences, not obtainable except in a specially-constructed building, should be enjoyed as soon as possible. The waste of labour is incredible, and although the patients are not allowed to suffer, the strain falls all the more heavily on the doctors and nurses. The hospital authorities undoubtedly do wisely in putting forth efforts to acquire a suitable building, not only from a selfish point of view, but because the maternity hospitals in the metropolis are inadequate to meet the needs of the respectable poor, that every bed placed at their disposal is a distinct gain to the community.

